# Where is the frontier between integrity in sport and anti-doping if it exists?

**DOI:** 10.3389/fspor.2023.1158055

**Published:** 2023-03-15

**Authors:** David Pavot, Raphael Faiss

**Affiliations:** ^1^ Research Chair on Anti-Doping in Sport, Business School, Department of Marketing, University of Sherbrooke, Sherbrooke, QC, Canada; ^2^ REDs, Research and Expertise in Anti-Doping Sciences, University of Lausanne, Lausanne, Switzerland

**Keywords:** doping, antidoping, integrity, sport, athlete

The World Athletics Federation founded the Athletics Integrity Unit in April 2017 in an effort to create the “right frameworks for each and every athlete to succeed.” More recently, the Anti-doping Switzerland Foundation was rebranded as Swiss Sport Integrity to provide a “fresh and modern image for a fair, clean, and credible sport.” This exemplifies a profound need to step back and give clean sport initiatives and anti-doping efforts a wider perspective.

Although the narrative of competition integrity has returned to the heart of sports in recent years, the issue is in fact not new. Indeed, cheaters were already pursued and sanctioned during ancient games, and some were even punished by notorious penalties. Thus, excavations in Olympia allowed, for example, the discovery of 16 pedestals of statues, known as the Zanes, located at the entrance of the stadium. These statues were erected with the money from the fines imposed by the judges on athletes violating the rules of the competitions, and their location was explicitly intended to deter any cheating attempt since any athlete, before entering the stadium, had to pass in front of the line of Zanes.

In the wake of the Festina scandal, the world of sporting events initially devoted itself to the fight against doping. WADA was established as a result, and the UNESCO-sponsored International Convention against Doping in Sport went into effect. Therefore, research capacity in the field has considerably strengthened in both biology and the social sciences. However, the pursuit of a dishonest-free sporting world cannot be restricted to doping. According to the UN, in 2021, there were 1,700 billion illegal bets on sporting events (1). Even within international sports federations, corruption cases have led to investigations and arrests. For example, as part of FIFAgate, nine FIFA officials were charged in 2015 at the request of the FBI (2). The national level is not exempt from criticism either. In 2022, Hockey Canada was at the center of a major scandal with the discovery of a federation fund to compensate victims of sexual abuse by national team players. This led to the creation of a Sport Integrity Commissioner (3). We also cannot ignore the allegations of sexual violence leveled against the president of the French Football Federation (4) or those that led to the resignation of the president of the French Handball Federation (5).

These repeated scandals have led to certain legislative or institutional changes. In some cases, the national anti-doping agency's scope of action has been broadened, as is the case with Sport Integrity Australia (6). In other cases, such as in Canada, parallel structures have emerged (2). Within international federations, following in the footsteps of athletics, integrity units are being developed that take a holistic approach to these problems. The question then arises: where is the frontier between integrity in sport and anti-doping, if it exists?

One may argue that anti-doping is obviously part of integrity, so anti-doping sciences necessarily fall under the “integrity” umbrella. While the latter makes sense, dealing with issues of inclusivity in athletes' participation in sporting events or trying to develop a new detection method for a forbidden substance ultimately requires very different expertise from various scientific angles.

After launching the Anti-doping section in Frontiers in Sports and Active Living in 2020, we underlined that education, deterrence, detection, enforcement, and the rule of law represent five firm pillars to delineate a broad field of exploration of doping and anti-doping initiatives with reference to the ever-evolving regulatory context of anti-doping (7). The focus was set on this “niche” section, while contributions to the journal and exchanges with the editors among the board made us realize that the notion of integrity in sports allowed us to redefine anti-doping research with input from numerous other disciplines.

This section has thus now evolved to be renamed “Anti-doping Sciences & Integrity in Sport” to broaden its scope and offer more researchers the chance to share their views and scientific research.

This evolution also follows the level of professionalization occurring in all sports, including not only athletes as professionals but also all their entourage, teams, or sponsors. Interestingly, the beam of light set on an individual cheater as a black sheep needs to be expanded to encompass all actors associated with any illegally enhanced athletic performance production. The World Anti-Doping Code, the main regulatory and harmonized document for anti-doping endorsed worldwide, has been revised in 2021 to better protect whistleblowers. These may indeed refer to “socially accepted” informants and reflect the need to sanction individuals trying to compromise the whistleblowers' integrity. Furthermore, the Code now also defines substances of abuse, recognizing that taking such substances [i.e., cocaine, methylenedioxymethamphetamine (MDMA), heroin, or tetrahydrocannabinol (THC)] may be indicative of a wider substance misuse or addiction problem with no related performance enhancement aim. The athlete is the center of attention, with the emphasis shifting away from the sole production of performance (and alleged illegal enhancement by doping) and back to their behavior, which is requested to be exemplary for the sake of the spirit of sport. This reflects the importance of athletic events as a social vector of (positive, but not exclusively so) emotions with underlying (and strong) moral principles.

Historically, there have been numerous professional athletes at the heart of financial shenanigans. Welsh track cyclist Jimmy Michael was undoubtedly the first athlete punished for a doping-related issue. He was considered one of the most successful and spectacular cyclists on the track in the late 1890s and was suspended in 1896 for alleged suspicious riding in a competition in London after claiming he had been drugged. Interestingly, he was not banned for the use of a forbidden substance or the fact that bets on that race were rigged, but only because he did not appear at his sporting federation's hearing. This example illustrates a professional athlete who was in a very lucrative circle of cyclists on the track at the center of an intricate mix of possibly corrupted officials, malicious entourages of competitors, and widespread use of various ergogenic mixtures for which he would not be tested. Corporate endorsements and sponsorships were the norm for the athletes' self-realization, and the social framework to define integrity was certainly more flexible. The Olympic movement then established amateurism as a standard to promote more fundamental educational and social values for sport. Nowadays, the spirit of sports is at the heart of the definition of doping, which therefore underpins the notion of integrity in this field.

Defending athletes who work hard and try their best by natural means seems odd in this context if one forgets that, aside from ethics, sporting federations and stakeholders need to tackle corruption, equality, and discrimination issues to safeguard the welfare of the athlete as a human being first.

While spectators are reassured to know that every medalist is tested for doping substances after the podium ceremony, it could be worth “conceptualizing doping as a sport integrity issue, to move away from the archaic and delimiting view of clean sport as drug-free sport,” as recently proposed by Petróczi and Boardley (8). Investigating the integrity of sporting organizations themselves may allow us to identify the gaps between their claims to strongly develop education programs for their athletes while only allocating a minor portion of their budget to that end.

The new name of the section is meant to be a premise rather than a choice. The remark is that, if we look at the modern history of sports after the end of the cold war, integrity has begun to be institutionally and substantially controlled by the fight against doping. The trend towards the development of integrity units in international federations, building on their expertise in anti-doping and moving towards other areas of integrity, seems to us to be very interesting. Also, perhaps one day WADA's remit will be extended along these lines, or perhaps we will see new regulators being created for gambling or even sexual violence. In any case, athletes deserve to be able to thrive in an environment of integrity, and this section intends to contribute to that.
